# Loneliness as neurobehavioral issue in amyotrophic lateral sclerosis

**DOI:** 10.1002/acn3.52028

**Published:** 2024-02-22

**Authors:** Monica Consonni, Veronica Faltracco, Eleonora Dalla Bella, Alessandra Telesca, Enrica Bersano, Anna Nigri, Greta Demichelis, Jean P. Medina, Maria G. Bruzzone, Giuseppe Lauria

**Affiliations:** ^1^ 3rd Neurology Unit and Motor Neuron Disease Centre Fondazione IRCCS Istituto Neurologico Carlo Besta via Luigi Celoria 11 Milan 20133 Italy; ^2^ Ph.D. Program in Neuroscience School of Medicine and Surgery, University of Milano‐Bicocca Via Cadore 48 Monza 20900 Italy; ^3^ Department of Medical Biotechnology and Translational Medicine University of Milan Via Vanvitelli 32 Milan 20133 Italy; ^4^ Neuroradiology Unit Fondazione IRCCS Istituto Neurologico Carlo Besta via Luigi Celoria 11 Milan 20133 Italy

## Abstract

**Objective:**

In elderly people loneliness represents a risk factor for dementia and may negatively impact on mental and physical health. The specific contribute of loneliness to cognitive and behavioral functioning have not yet been determined in amyotrophic lateral sclerosis (ALS). Our hypothesis was that loneliness may be related to motor dysfunction with a negative impact on cognitive and behavioral decline, possibly related to specific cortical involvement.

**Methods:**

In 200 ALS patients (ALSpts) and 50 healthy controls (HCs) we measured loneliness, mood, and quality of life (QoL). ALSpts underwent comprehensive clinical, genetic, and neuropsychological assessment to define phenotypes. Seventy‐seven ALSpts performed 3T MRI scans to measure cortical thickness. Between‐group, partial correlation and regression analyses were used to examined clinical, neuropsychological, and cortical signatures of loneliness.

**Results:**

Feelings of loneliness were documented in 38% of ALSpts (ALS/L+pts) and in 47% of HCs. In both groups loneliness was associated with anxiety (*P* < 0.001), depression (*P* ≤ 0.005), and poor QoL (*P* < 0.001). ALS/L+pts had similar motor dysfunctions and cognitive abilities than non‐lonely ALSpts, but distinct behavioral profiles (*P* ≤ 0.005) and frontoparietal involvement (*P* < 0.05). Loneliness in ALS is related to behavioral changes, apathy, and emotional dysregulation (*P* < 0.001).

**Interpretation:**

Our cross‐sectional study indicates that, in ALS, the satisfaction of social environment is associated with a sense of life well‐being that is not limited to the motor status, proving instead that loneliness can impact on disease‐related neurobehavioral changes with a possible flashback on brain architecture. This suggests that sociality could promote personal resilience against behavioral and affective decline in ALS.

## Introduction

Loneliness is a unique condition that gives an individual the feeling of being socially isolated. One can feel lonely even when surrounded by other people, as loneliness depends on satisfaction of one's social relationships.^1^ Being linked to several indicators of poor psychological well‐being, cognitive impairment, depression, and somatic symptoms, loneliness is currently considered a risk factor for broad‐based morbidity in human and animal models.^2^ In aging, the richness and satisfaction of social connections and support might protect against dementia by motivating health behaviors that can help effective use of available health services or by acting as a buffer against stress and thereby protect against dementia.^3^ The strength of the associations between poor perceived social interaction and incident dementia is comparable with other well‐established risk factors for dementia, including low education, genetic variables, and late‐life depression,^4^ indicating possibly associated neurobiological mechanisms. In this viewpoint, recent studies showed that the experience of loneliness may alter the structure and function of the prefrontal cortex, especially medial and dorsolateral regions, anterior insula, amygdala, hippocampus, and posterior superior temporal cortex.^5^

Our study sought to shed light on how loneliness impacts on people with amyotrophic lateral sclerosis (ALS), a neurodegenerative disease leading to progressive motor impairment and disability and, in half of the cases, cognitive decline and behavioral changes (apathy, disinhibition, and emotional blunting).^6^ Some studies have investigated the association of loneliness and mental health in ALS and their caregivers,^7^ suggesting a strong relationship between perceived social isolation, depression, anxiety, and wish to hasten death.^8^ However, the specific contribute of loneliness to cognitive and behavioral functioning and cortical networks have not yet been determined in ALS. To this aim, we investigated the degree of loneliness feelings in ALS and its correlations with clinical and neuropsychological profiles, and cortical involvement.

## Material and Methods

### Participants

Between January 2017 and June 2021, consecutive patients diagnosed with ALS based on the revised El Escorial criteria were enrolled. Patients with first language other than Italian, clinical and imaging evidence of cerebrovascular disease, neurocognitive developmental disorder, primary psychiatric disorders and forced vital capacity <70% were excluded. Fifty volunteers were enrolled as healthy control group (HC) without history of neurological disease and primary psychiatric disorder.

Each participant was enrolled after giving written informed consent. Participant's consent was obtained according to the Declaration of Helsinki. The database was anonymized according to the Italian law for the protection of privacy. The study was approved by the local Ethic Committee.

### Clinical assessment

Loneliness was measured using the 3‐item version of the University of California, Los Angeles Loneliness Scale (UCLA‐3L, range: 0–6 points)^9^ (Table 1). Self‐reports of mood (Hospital Anxiety and Depression Scale for motor neuron disease ‐ HADS‐MND^10^; Beck Depression Inventory ‐ BDI‐II), alexithymia (20‐item Toronto Alexithymia Scale, TAS‐20),^11^ and quality of life (QoL)^12^ were administered to all participants. In patients, cognitive efficiency and social cognition were assessed with the Edinburgh Cognitive and Behavioral ALS Screen (ECAS),^13^ the Story‐based Empathy Task (SET),^14^ the Ekman 60‐Faces Test^15^ and the Difficulty in Emotional Regulation (DERSF) scale.^16^ Anxiety was measured with the Spielberger's State‐Trait Anxiety Inventory (STAIY‐1 and STAIY‐2). Behavioral profiles of patients were assessed by means of semi‐structured clinical interviews with patients' family members. Patients' behavioral changes were measured with the Frontal Behavioral Inventory‐ALS version (FBI‐ALS).^17^ The caregiver‐rated Dimensional Apathy Scale (DAS) was used to evaluate the emotional, executive and cognitive/behavioral initiation subtypes of apathy.^18^ All patients with cognitive (ALSci), behavioral (ALSbi), combined cognitive and behavioral (ALScbi) and/or frontotemporal dementia‐like (ALS‐FTD) impairment were defined according to the recent guidelines.^6^ The burden of patients' family members was addressed with the Caregiver Burden Inventory (CBI).^19^

**Table 1:** Differences in demographical and psychometric measures of ALS and HC participants. Spearman rho correlations (Bootstrapping confidence intervals) between UCLA loneliness scale – 3 Item version (UCLA‐3L) scores and measures are reported for both groups.

	ALS			HC			Comparisons	Correlations
	No.	Range	Mean (SD)	No.	Range	Mean (SD)	*Z* (Sig.)	Spearman *ρ* (CIs); Sig
**Demographical data**								
Gender (male/female)	200	–	101/99	49	–	20/29		–
Years of age	200	22–88	59.74 (11.7)	49	45–73	60.02 (8.6)	−0.32 (0.750)	ALS: 0.121; *P* = 0.088 HC: −0.019; *P* = 0.902
Years of education	200	5–25	12.79 (3.8)	45	5–23	13.26 (4.1)	−0.63 (0.527)	**ALS: −0.194 (−0.32 to −0.06); *P* = 0.006** HC: 0.117; *P* = 0.449
**Loneliness**								
UCLA‐3L total score	200	3–9	3.85 (1.3)	49	3–5	3.95 (1.5)	−1.27 (0.202)	–
UCLA‐3L item 1	200	1–3	1.36 (0.5)	49	1–2	1.42 (0.6)	−0.59 (0.550)	–
UCLA‐3L item 2	200	1–3	1.23 (0.5)	49	1–2	1.38 (0.6)	−1.86 (0.066)	–
UCLA‐3L item 1	200	1–3	1.22 (0.5)	49	1–2	1.30 (0.5)	−1.44 (0.149)	–
**Psychometric values**								
Anxiety (HADS‐MND subscore)	194	0–17	5.32 (3.6)	49	0–12	4.89 (3.1)	−0.57 (0.567)	**ALS: 0.299 (0.16 to 0.41); *P* < 0.001** **HC: 0.508 (0.25 to 0.71); *P* < 0.001**
Depression (HADS‐MND subscore)	194	0–18	2.78 (2.7)	49	0–8	2.77 (2.2)	−0.42 (0.672)	**ALS: 0.400 (0.26 to 0.51); *P* < 0.001** HC: 0.162; *P* = 0.265
Beck depression inventory	171	0–50	9.19 (7.7)	49	0–28	8.08 (7.0)	−1.02 (0.308)	**ALS: 0.464 (0.33 to 0.57); *P* < 0.001** **HC: 0.391 (0.12 to 0.61); *P* = 0.005**
Quality of life(WhoQol‐Age)	184	1–5	3.48 (0.6)	49	2–5	3.90 (0.5)	**−3.831 (0.001)**	**ALS: −0.391 (−0.50 to −0.27); *P* < 0.001** **HC: −0.383 (−0.60 to −0.14); *P* = 0.007**
Alexithymia (TAS)	167	17–78	44.57 (11.9)	34	27–63	44.73 (10.0)	−0.16 (0.867)	**ALS: 0.303 (0.15 to 0.44); *P* < 0.001** **HC: 0.443 (0.09 to 0.68); *P* = 0.009**

Significant differences and correlations surviving Bonferroni correction are reported in bold. HADS‐MND, Hospital Anxiety and Depression Scale for use in motor neuron disease; TAS, Toronto Alexithymia Scale; WhoQol‐Age, The World Health Organization Quality of Life in the Aging population.

Type of disease‐onset, disease duration, motor disability (revised ALS Functional Rating Scale, ALSFRS‐R) and disease stage (King's Clinical Staging System, KCSS) was documented for all patients.

### Genetic analysis

DNA was extracted from peripheral blood. Next‐generation sequencing analysis was performed by means of amplicon deep sequencing using Sure Select QXT kit (Agilent) for ALS causative genes (SOD1, FUS, TARDBP, VCP, OPTN, SQSTM1, TUBA4A, PFN1, and UBQLN2) and repeat primed PCR AmplideX PCR/CE C9ORF72 kit (Asuragen Inc., Austin TX) for detection of C9orf72 expansion.

### MRI data analysis

A subgroup of 77 patients performed MRI. Imaging data were acquired using an Achieva 3T MRI scanner (Philips Healthcare NL) equipped with a 32‐channel head coil. The MRI protocol included a high‐resolution 3D T1‐weighted (TR = 8.3 ms, TE = 3.8 ms, FOV = 240 × 240 mm, no gap, voxel size = 1 × 1 × 1 mm^3^, flip angle = 8°, 180 sagittal slices) and 3D fluid‐attenuated inversion recovery (TR = 5000 ms, TI = 1700 ms, TE = 300 ms, FOV = 240 × 240 mm, no gap, voxel size = 1 × 1 × 1 mm^3^, 180 sagittal slices) sequences. The images were examined by neuroradiologists to exclude incidental findings in study participants. Cerebral cortex thickness (CT) was evaluated using FreeSurfer software (http://freesurfer.net, version 6). The “recon–all” pipeline was applied using both T1‐weighted and FLAIR images to improve the pial surface reconstruction.^20^, ^21^, ^22^ The mean CT of each region according to Desikan‐Killiany atlas parcellation was obtained.^23^ A subset of regions of interest within the “social brain network” or closely related to the processing of social stimuli and social behaviors were selected from those previously extracted. See Supplementary Material for details.

### Statistical analyses

Comparison between ALS and HC groups in demographical and psychometric data were assessed using the Mann‐Whitney *U*‐tests. The association of loneliness with demographical and neuropsychological data were assessed with Spearman's rho correlations with Bootstrapping confidence intervals. Differences in clinical and demographical variables between ALS patients with feelings of loneliness (**ALS/L+**; UCLA‐3L≥4) and patients without feeling of loneliness (**ALS/L−**; UCLA‐3L= 3) were examined with independent‐sample *t* tests and chi‐squared exact tests. Variables reaching significant (*P* < 0.05) between‐subgroup differences were used as confounding variables for the subsequent analyses of variance. The one‐way ANOVA was used to explore differences in ALS subgroups on psychometric data with years of age, years of education, and ALSFRS‐R scores as covariates with statistical significance determined at *P* ≤ 0.05 after Bonferroni correction for multiple comparisons. In addition, chi‐squared exact tests were performed to verify the co‐occurrence of feelings of loneliness (number of patients with UCLA‐3L score ≥4) and cognitive impairment (ALSci, ALScbi, and ALS‐FTD), social cognition deficits (pathological performances at SET and Ekman tests), behavioral impairment (ALSbi, ALScbi, and ALS‐FTD), symptoms of apathy (FBI‐ALS apathy subscore ≥1), anxiety (HADS‐MND subscore ≥8), and depression (HADS‐MND subscore ≥7). Partial correlations (controlling for the effect of age, education, ALSFRS‐R, and HADS‐MND total score) examined factors mainly associated with loneliness in all patients. We included mood as confounding variable in the view of the high association with loneliness. Bonferroni correction for multiple correlation analyses was applied. All significant correlations were bias corrected, and accelerated bootstrap 95% intervals were computed with 1000 bootstrap equally sized samples obtained. Hierarchical regressions were then performed to confirm the role of loneliness, over age and disease stage, on psychometric variables significantly associated with loneliness in ALS patients: age and KCSS stage were included in the model first (Step 1) and then UCLA‐3L scores (Step 2).

One‐way ANOVA was used to explore differences in CT of brain regions of **ALS/L+** compared to **ALS/L−** patients, using age as confounding variable. Partial correlations further examined the association of CT with loneliness in all patients, controlling for the effect of age, and ALSFRS‐R and HADS‐MND total scores. Additionally, hierarchical regression analyses were then carried out to test if loneliness, age, KCSS stage, behavioral (FBI‐ALS scores), and cognitive functioning (ECAS scores corrected for age and education according to the Italian regression‐based norms; corrECAS^24^) can predict CT in brain regions showing significant between‐group differences or correlations. Age and KCSS stages were included in the model first (Step 1), then FBI‐ALS and corrECAS (Step 2) and UCLA‐3L was entered in the model as final step (Step 3). In the view of the explorative analysis, *P* < 0.05 were taken as significant. All statistical tests were two‐tailed. Statistical analyses were performed using SPSS (IBM SPSS statistics version 21).

## Results

Two‐hundred and seventy‐two ALS patients underwent clinical and neuropsychological examination. Of these, 72 were excluded from the study: 58 patients did not complete the UCLA‐3L, 10 had MRI evidence of cerebrovascular disease; 2 had psychiatric disorders (major depression, post‐traumatic stress disorder); 1 had neurocognitive developmental disorder; and 1 was not compliance to the cognitive assessment. We further excluded one volunteer with BDI score above cutoff for severe depressive mood.

### Loneliness in ALS and HC

**Figure 1:**
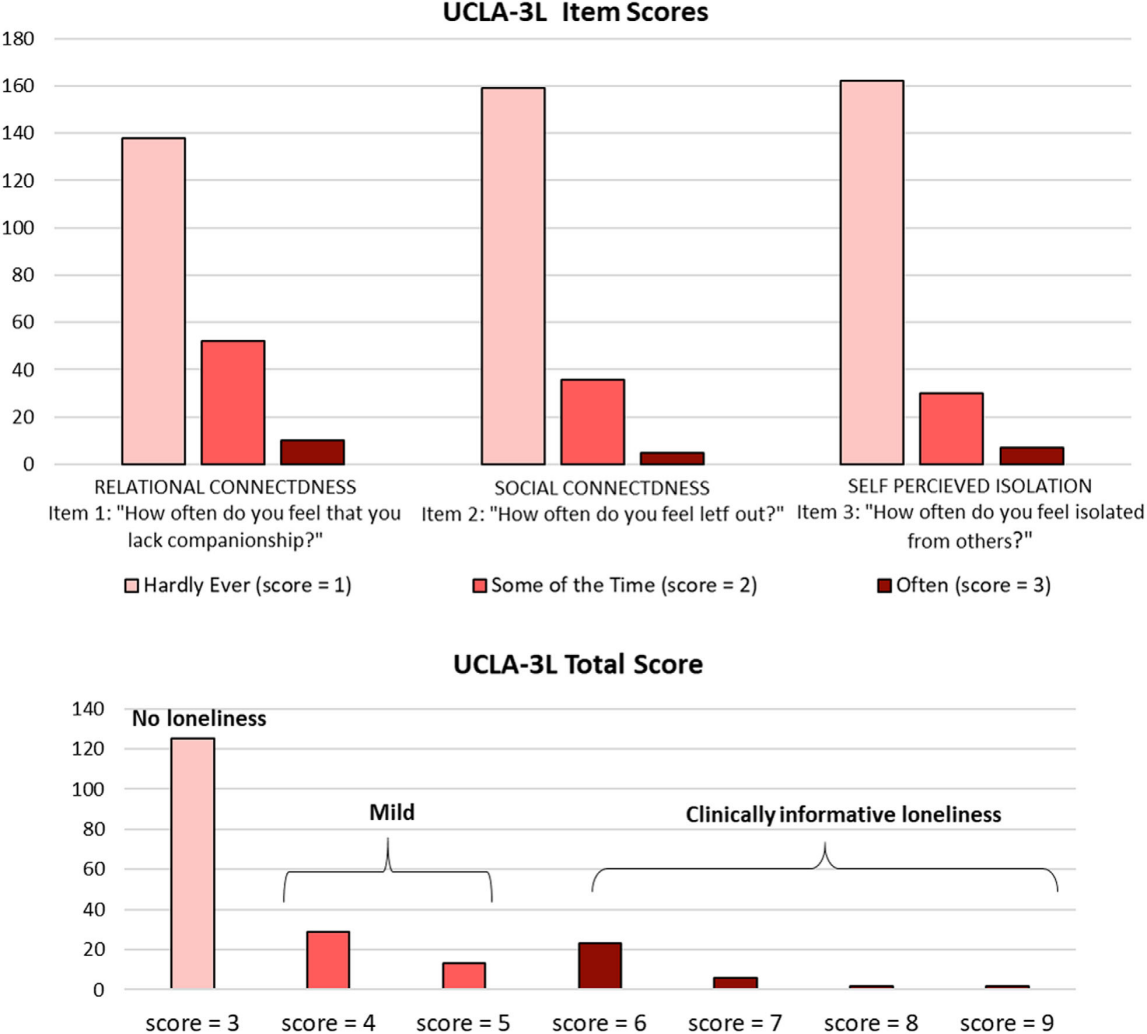
Scoring of the 3‐item version of the University of California, Los Angeles loneliness scale (UCLA‐3L) in a sample of 200 patients with ALS.

Of the 200 patients enrolled, 125 (62%) patients did not have feeling of loneliness (**ALS/L−;** UCLA‐3L=3), 65 (32%) had mild feeling of loneliness (UCLA‐3L ranging from 4 to 5), and 33 (16%) obtained cutoff score (UCLA‐3L≥6) suggestive of clinically informative loneliness (see Fig. 1). Among HC, 26 volunteers (53%) did not have loneliness, 12 (25%) had mild loneliness, and 11 (22%) had UCLA‐3L≥6. The degree of loneliness was similar in the two groups (*χ* ^2^ = 2.56, *P* = 0.278). ALS and HC participants had comparable psychological profile, but QoL that was lower in ALS than HC. In both groups, loneliness was associated with depressive symptoms, anxiety levels, alexithymia, and reduced QoL (Table 1).

### Neuropsychological profiles of ALS/L+ and ALS/− patients

Results of subgroup comparisons are reported in Table 2. ALS/L+ and ALS/L− patients had similar clinical phenotypes, but the occurrence of cognitive and/or behavioral impairment was more frequent in ALS/L+ than ALS/L−. Because of between‐group differences, albeit not surviving Bonferroni correction, years of age, years of education, and ALSFRS‐R scores were used as covariates in the analyses of variance showing that ALS/L+ had more severe apathetic behavior, higher symptoms of anxiety, depression, emotional dysregulation, alexithymia, and reduced QoL than ALS/L−.

**Table 2:** Differences in clinical and neuropsychological measures and partial correlations between the UCLA loneliness scale – 3 Item version (UCLA‐3L) scores and clinical/neuropsychological measures of ALS patients with (ALS/L+) and without feeling of loneliness (ALS/L−).

	No.	Range	ALS/L−	No.	Range	ALS/L+	Comparisons (Sig.)	Partial correlations (Sig.)^1^
**Loneliness (UCLA‐3L)**	125	3	3 (0)	75	4–9	5.26 (1.2)	–	–
**Demographic and clinical data**								
Gender (male/female)	125	–	60/65	75	–	41/34	*χ* ^2^ = 0.833 (0.361)	–
Years of age	125	22–88	58.27 (12.1)	75	30–86	62.0 (10.6)	**T = −2.158 (0.032)**	0.008 (0.909)^3^
Years of education	125	5–25	13.39 (3.9)	75	5–18	11.79 (3.3)	**T = 2.936 (0.004)**	−0.080 (0.684)^4^
Months from symptom onset	119	4–84	21.27 (17.2)	75	2–72	19.70 (14.7)	T = 0.483 (0.629)	0.003 (0.967)
Motor disability (ALSFRS‐R)	124	19–47	40.89 (4.8)	74	24–47	39.33 (4.9)	**T = 2.153 (0.033)**	−0.118 (0.103)^5^
King's clinical stage (1/2/3/4)	125	1–4	57/43/23/2	75	1–4	23/30/21/1	*χ* ^2^ = 5.002 (0.161)	–
Bulbar onset (yes/no)	125	–	21/104	75	–	18/57	*χ* ^2^ = 1.587 (0.213)	–
Gene mutations (yes/no)	125	–	19/105	75	–	18/57	*χ* ^2^ = 0.060 (0.805)	–
C9orf72/SOD1/others/n.a.		–	12/3/4/1		–	6/3/4/0		–
ALScn/ALSimp	125	–	67/58	75	–	28/47	***χ*** ^2^ **= 4.974 (0.025)**	–
**Psychometric data** ^2^								
Cognitive efficiency (ECAS)	122	59–127	106.28 (13.5)	66	44–125	103.1 (15.0)	*F* = .001 (0.970)	0.030 (0.684)
Empathy (SET total score)	81	7–18	14.25 (2.7)	56	3–18	13.05 (3.5)	*F* = 1.132 (0.252)	−0.067 (0.448)
Emotion recognition (Ekman test)	84	14–57	45.95 (6.8)	42	14–53	44.14 (6.9)	*F* = 0.544 (0.462)	−0.177 (0.051) [−0.32 to 0.02]
Emotional regulation (DERS)	109	36–138	60.20 (13.8)	64	41–138	77.01 (20.5)	***F* = 32.708 (<0.001)**	**0.280 (<0.001) [0.14 to 0.42]**
Behavioral change (FBI‐ALS)	101	0–28	2.28 (2.3)	61	0–28	5.15 (6.4)	*F* = 7.941 (0.005)	**0.320 (<0.001) [0.08 to 0.54]**
Apathy (DAS)	90	4–46	17.92 (8.1)	56	5–50	25.44 (10.9)	***F* = 21.389 (<0.001)**	**0.406 (<0.001) [0.20 to 0.56]**
Anxiety (HADS‐MND)	125	0–12	4.56 (3.2)	70	0–17	6.67 (3.8)	***F* = 14.258 (<0.001)**	**0.300 (<0.001)** ^6^ **[0.18 to 0.41]**
State anxiety (STAIY‐1)	105	22–68	41.73 (8.33)	63	32–74	46.61 (8.35)	***F* = 13.306 (<0.001)**	0.095 (0.230)
Trait anxiety (STAIY‐2)	106	26–58	38.6 (6.39)	61	28–71	45.93 (8.62)	***F* = 32.515 (<0.001)**	**0.321 (<0.001) [0.17 to 0.47]**
Depression (HADS‐MND)	125	0–11	2.00 (1.9)	70	0–18	4.18 (3.34)	***F* = 25.833 (<0.001)**	**0.327 (<0.001)** ^6^ **[0.19 to 0.46]**
Depression (BDI‐II)	109	0–23	6.57 (4.87)	62	0–50	13.7 (9.56)	***F* = 34.596 (<0.001)**	**0.431 (<0.001) [0.26 to 0.56]**
Alexithymia (TAS)	102	17–78	42.1 (10.6)	61	17–78	48.70 (12.9)	***F* = 7.134 (0.008)**	0.143 (0.074)
Quality of life (WhoQol‐Age)	118	1–5	3.65 (0.5)	66	1–5	3.17 (0.6)	***F* = 21.012 (<0.001)**	**−0.243 (0.001) [−0.35 to −0.12]**
Caregiver burden (CBI)	79	0–40	8.13 (9.1)	50	0–50	10.94 (11.4)	*F* = 2.644 (0.107)	0.254 (0.005) [0.04 to 0.45]

Significant differences and correlations surviving Bonferroni correction are reported in bold. ALScn, cognitively normal ALS; ALSFRS‐r, revised ALS Functional Rating Scale; ALSimp, ALS with cognitive and/or behavioral impairment; BDI‐II, Beck depression scale; CBI, Caregiver Burden Inventory; DAS, Dimensional Apathy Scale; DERSF, Difficulties in Emotion Regulation Scale; ECAS, Edinburgh Cognitive and Behavioral ALS Screen; FBI‐ALS, Frontal Behavioral Inventory – ALS version; HADS‐MND, Hospital Anxiety and Depression Scale for use in motor neuron disease; SET = Story‐based Empathy Task; STAIY, Spielberger's State–Trait Anxiety Inventory; TAS, Toronto Alexithymia Scale; WhoQol‐Age , The World Health Organization Quality of Life in the Aging population.

^1^ Correction for age, education, HADS‐MND and ALSFRS‐R.

^2^ ANOVA with age, education, and ALFSRS‐R as covariates.

^3^ Correction for education, HADS‐MND and ALSFRS‐R.

^4^ Correction for age, ALSFRS‐R and HADS‐MND.

^5^ Correction for age, education, and HADS‐MND.

^6^ Correction for age, education, and ALSFRS‐R.

Partial correlation analyses documented that mood, behavioral changes, emotional dysregulations, and poor QoL were significantly related to loneliness (Table 2). Contingency table revealed that the occurrence of loneliness was significantly associated with depression, diagnosis of ALSbi, and apathy (Table 3).

**Table 3:** Contingency tables (percentages enclosed in parentheses) of the co‐occurrence of loneliness (UCLA‐3L score ≥1) and specific neuropsychological performances.

**Cognition**	**Cognitive impairment** ^1^	**Normal profile**	Total	*χ* ^2^ = 0.0889 *P* = 0.765
UCLA‐3L≥4	29 (14.5%)	46 (23%)	75 (37.5%)	
UCLA‐3L=3	51 (25.5%)	74 (37%)	125 (62.5%)	
Total	80 (40%)	120 (60%)	200 (100%)	
**ECAS performances**	**Below cutoff** ^2^	**Above cutoff**	Total	*χ* ^2^ = 0.1668 *P* = 0.682
UCLA‐3L≥4	15 (7.9%)	51 (27.1%)	66 (35.1%)	
UCLA‐3L=3	31 (16.4%)	91 (48.4%)	122 (64.8%)	
Total	46 (24.4%)	142 (75.5%)	188 (100%)	
**SET performances**	**Below cutoff**	**Above cutoff**	Total	*χ* ^2^ = 0.2273 *P* = 0.633
UCLA‐3L≥4	10 (7.2%)	46 (33.5%)	56 (40%)	
UCLA‐3L=3	12 (8.7%)	69 (50%)	81 (59.1%)	
Total	22 (16%)	115 (83.9%)	137 (100%)	
**Ekman test performances**	**Below cutoff**	**Above cutoff**	Total	*χ* ^2^ = 0.1064 *P* = 0.744
UCLA‐3L≥4	7 (5.5%)	35 (27.7%)	42 (33.3%)	
UCLA‐3L=3	16 (12.6%)	68 (53.9%)	84 (66.6%)	
Total	23 (18.6%)	103(81.7%)	126 (100%)	
**Behavior**	**Behavioral impairment** ^3^	**Normal profile**	Total	*χ* ^2^ = 12.7734 *P* < 0.001
UCLA‐3L≥4	28 (14%)	47(23.5%)	75 (37.5%)	
UCLA‐3L=3	19 (9.5%)	106 (53%)	125 (62.5%)	
Total	47 (23.5%)	153 (76.5%)	200 (100%)	
**Symptoms of apathy**	**Presence of symptoms** ^4^	**Absence of symptoms**	Total	*χ* ^2^ = 10.8701 *P* < 0.001
UCLA‐3L≥4	32 (19.7%)	29 (17.9%)	61 (37.6%)	
UCLA‐3L=3	27 (16.6%)	74 (45.6%)	101 (62.3%)	
Total	59 (36.4%)	103 (63.5%)	162 (100%)	
**Depression**	**Presence of depression** ^5^	**Absence of depression**	Total	*χ* ^2^ = 10.8334 *P* < 0.001
UCLA‐3L≥4	22 (11.3%)	48 (24.7%)	70 (36%)	
UCLA‐3L=3	15 (7.7%)	109 (56.1%)	124 (63.9%)	
Total	37 (19%)	157 (80.9%)	194 (100%)	

The *P*‐values of chi‐squared tests are reported.

^1^ Cognitive impairment was diagnosed according to the Strong et al. criteria (2017) on the basis of the occurrence of either executive dysfunction, including social cognition, or language dysfunction or a combination of the two.

^2^ ECAS total scores below the Italian norms defined by Poletti et al. (2016).

^3^ Behavioral impairment was diagnosed according to the Strong et al. criteria (2017) on the basis of semi‐structured clinical interviews with patients' adult family members.

^4^ Symptoms of apathy were attributed to patients if the apathy subscore of the frontal behavioral inventory (FBI‐ALS) was ≥1.

^5^ Depression was attributed to patients obtaining a score above cutoff (≥ 8) at the depression‐subscore of Hospital Anxiety and Depression Scale for motor neuron disease (HADS‐MND).

Regression analyses confirmed the role of loneliness, older age, and disease stage on neurobehavioral changes in ALS (Table 4). We found that ALS/L+ patients had higher scores in all DAS subdomains than ALS/L− patients and that UCLA‐3L scores were significantly associated with all DAS subscores (Table S1). A positive correlation was also found between UCLA‐3L and DERS subscores assessing the limited use of emotion regulation strategies and the non‐acceptance of emotional responses (Table S2).

**Table 4:** Final linear models evidencing significant predictors.

Dependent variables	Constant and independent variables	B (confidence intervals)	Bootstrapped standard error *B*	*β*	Bootstrapped *P*‐value (two‐tailed)
**FBI‐ALS** *R* = 0.413, adjusted *R* ^2^ = 0.154 *F*_3.158_ = 10.609; *P* < 0.001	Constant	1.343 (−2.831 to 5.493)	2.029	–	0.530
	Age	−0.016 (−0.094 to 0.060)	0.040	−0.033	0.701
	King's stage	0.896 (−0.344 to 1.984)	0.575	0.138	0.130
	**UCLA**	1.455 (0.585 to 2.379)	0.469	0.379	0**.008**
**DAS – apathy score TOT** *R* = 0.413, adjusted *R* ^2^ = 0.154 *F*_3.158_ = 10.609; *P* < 0.001	Constant	20.235 (13.144 to 26.849)	3.872	–	0.001
	Age	−0.092 (−0.242 to 0.060)	0.065	−0.104	0.172
	King's stage	1.698 (‐0.385 to 3.592	1.049	0.141	0.101
	**UCLA**	3.086 (2.019 to 4.194)	0.569	0.445	**0.001**
**HADS‐ depression score** *R* = 0.364, adjusted *R* ^2^ = 0.119 *F*_3.193_ = 9.652; *P* < 0.001	Constant	0.849 (−0.854 to 2.595)	0.904	–	0.356
	Age	0.024 (−0.007 to 0.055)	0.015	0.102	0.116
	King's stage	−0.036 (−0.494 to 0.370)	0.233	−.011	0.8786
	**UCLA**	0.714 (0.397 to 1.095)	0.182	0.343	**0.001**
**HADS‐ anxiety score** *R* = 0.311, adjusted *R* ^2^ = 0.083 *F*_3.193_ = 6.183; *P* < 0.001	Constant	4.342 (1.724 to 6.863)	1.272	–	0.002
	Age	0.004 (−0.033 to 0.044)	0.019	0.012	0.838
	King's stage	0.049 (−0.527 to 0.612)	0.312	0.011	0.876
	**UCLA**	0.837 (0.505 to 1.229)	0.177	0.308	**0.001**
**STAY‐2 trait anxiety score** *R* = 0.481, adjusted *R* ^2^ = 0.217 *F*_3.166_ = 16.351; *P* < 0.001	Constant	37.965 (32.028 to 43.266)	2.910	–	0.001
	Age	0.002 (−0.086 to 0.105)	0.048	0.003	0.957
	King's stage	.451 (−0.807 to 1.678)	0.675	0.046	0.496
	**UCLA**	2.859 (1.916 to 4.039)	0.481	0.473	**0.001**
**DERS‐F** *R* = 0.453, adjusted *R* ^2^ = 0.191 *F*_3.172_ = 14.540; *P* < 0.001	**Constant**	56.001 (45.633 to 65.967)	5.527	–	0.001
	Age	.033 (−0.164 to 0.240)	0.097	0.021	0.741
	King's stage	1.944 (−1.283 to 5.085)	1.603	0.087	0.240
	**UCLA**	5.980 (3.757 to 8.555)	1.206	0.429	**0.001**
**TAS‐20** *R* = 0.328, adjusted *R* ^2^ = 0.091 *F*_3.162_ = 6.383; *P* < 0.001	Constant	38.227 (29.844 to 45.523)	4.047	–	0.001
	Age	0.022 (−0.113 to 0.171)	0.071	0.022	0.747
	King's stage	1.534 (−0.814 to 3.713)	1.108	0.105	0.159
	**UCLA**	2.577 (1.227 to 3.843)	0.690	0.292	**0.002**
**QoL – Eurohis** *R* = 0.474, adjusted *R* ^2^ = 0.212 *F*_3.181_ = 17.199; *P* < 0.001	Constant	32.680 (28.890 to 36.803)	1.941	–	0.001
	Age	−0.024 (−0.086 to 0.033)	0.031	−0.055	0.441
	**King's stage**	−1.108 (−1.895 to −0.299)	0.425	−0.177	**0.015**
	**UCLA**	−1.542 (−2.103 to −1.075)	0.273	−0.400	**0.001**

Significant predictors are reported in bold. DAS, Dimensional Apathy Scale; DERSF, Difficulties in Emotion Regulation Scale; FBI‐ALS, Frontal Behavioral Inventory – ALS version; HADS‐MND, Hospital Anxiety and Depression Scale for use in motor neuron disease; STAIY, Spielberger's State–Trait Anxiety Inventory; TAS, Toronto Alexithymia Scale; QoL – Eurohis, The World Health Organization Quality of Life in the Aging population.

### Cortical thickness correlates of loneliness in ALS

**Figure 2:**
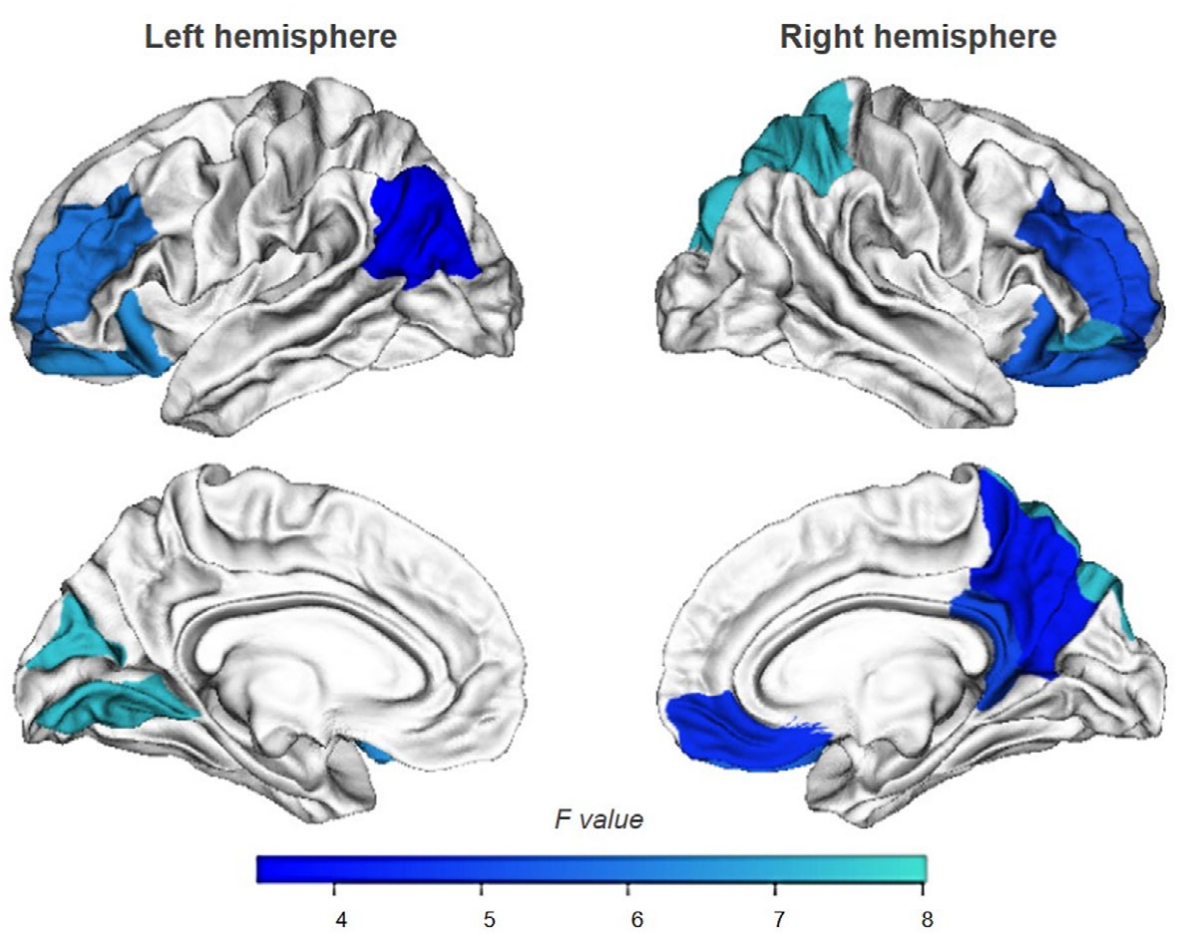
Brain regions showing cortical thinning in ALS patients with feelings of loneliness (ALS/L+) compared to non‐lonely ALS patients (ALS/L−). For exploratory analysis, the results are reported at a threshold of *P* < 0.05 uncorrected for multiple comparisons.

Demographical, clinical, and neuropsychological data of ALS patients (*N* = 77) undergoing MRI are reported in Table S3. In this group, 32 patients (41%) had UCLA‐3L≥4 (ALS/L+), 45 had no feelings of loneliness (ALS/L−). Between‐group and partial correlation analyses indicated that the neuropsychological profile of the ALS/L+ and ALS/L− patients performing MRI scans did not differ from those of the whole cohort of ALS patients. Consistently, we did not find significant differences on overall demographical, clinical and neuropsychological data of patients with and without MRI scans (*P* > 0.10). Interestingly, ALS/L+ patients had reduced CT in bilateral frontoparietal cortices and left occipital regions compared to ALS/L− patients (Fig. 2). Partial correlations showed that CT was not related to loneliness (Table S4), but regression analyses revealed that thinning of the right precuneus was significantly associated with loneliness, older age, and behavioral changes (Table S5).

## Discussion

Using a validate scale largely used to assess perceived states of social and emotional isolation,^9^ we measured to what extend ALS patients, whose progressive motor dysfunctions could limit social activities, may feel lonely. Our hypothesis was that loneliness in ALS may be related to motor dysfunction with a negative impact on cognitive and behavioral profiles possibly related to specific cortical involvement. Surprisingly, we found that ALS patients had similar feeling of loneliness than HC and that loneliness was not related to functional motor status or cognitive abilities. We found instead that loneliness was associated with behavioral changes and affective states.

### Neurobehavioral changes and loneliness in ALS

Loneliness represents a risk factor for a broad‐base mental and physical health problems^2^ and, although the prevalence of loneliness varies with age, around 15–30% of adults report chronic or severe loneliness.^25^, ^26^ Consistently, we found that about 38% of ALS patients and 47% of HC volunteers felt somewhat‐to‐moderately lonely, but only 16% of ALS and 22% of HC participants obtained scores suggesting that loneliness can be clinically informative as risk for mental health problems (UCLA‐3L≥6). In line with literature data on older adults, in both ALS and HC groups, the higher the loneliness the more severe the symptoms of depression and anxiety and the lower the QoL, suggesting that loneliness may be independent of physical health, and mainly related to mental health.^27^

Consistently, the feelings of social isolation in patients were neither related to motor disabilities nor disease duration, and were similar in those with bulbar and spinal onset, as well as in patients with or without genetic mutations (Table 2). Loneliness was not even found to be associated with the stage of the disease. This suggests that their satisfaction of social environment was associated with a sense of life well‐being that was not limited to the functional motor status or the time of illness. Previous reports showed that the majority of ALS patients and caregivers experienced the loss of participation in regular activities, feelings of being “homebound” and disconnected from the community as disease progress.^28^ These changes in social connections and reduced social activities^7^, ^28^ were associated with feelings of loneliness, poor QoL, severe depressive mood, and emotional distress.^8^, ^29^ In our sample, regression analyses documented the pivotal role of loneliness, over age and disease stage, on depressive symptoms of ALS patients (Table 4). In a recent longitudinal study (up to 12 years of observations), loneliness was confirmed to be a risk factor for developing depression.^30^ However, loneliness can also be a symptom of depression itself,^31^ and consistently 60% of ALS patients with depressive mood had feelings of loneliness. However, in our ALS cohort, depression had low incidence (31%) (Table 3), suggesting that, at least in some patients, loneliness and depression might occur independently.

We also found a strong association between loneliness and apathy (Tables 2 and 4), a behavioral symptom that is common in ALS.^6^ The prevalence of loneliness was higher among patients with symptoms of apathy (54%) than in non‐apathetic patients (28%) (Table 3). As apathy is established as a multidimensional construct, we used a scale (accounting for physical disability), that is, the DAS, measuring different subtypes of apathy: executive (lack of motivation for planning, attention, or organization), emotional (indifference, affective or emotional neutrality, flatness, or blunting), and initiation (lack of motivation for self‐generation of thoughts and/or actions).^18^ We found that 15% of patients had abnormal levels of apathy on at least one subscale, showing that ALS/L+ patients presented mainly greater difficulties in organizing thoughts and actions, attention, and planning (executive apathy) than ALS/L− patients. Correlation analyses indicated a strong relationship between loneliness and the reduced emphatic responses to emotional items (emotional apathy subtype) (Table S1). This significant positive association reinforces the link between social withdrawal and perceived social distance. Accordingly, in a large sample of community‐dwelling older persons with no comorbid depressive symptoms or cognitive impairment, apathy contributed to the perception of social isolation reducing their QoL.^32^ But, in a cross‐sectional study on patients with dementia, UCLA loneliness scale scores were not related to the apathy subscale of the Neuropsychiatric Inventory.^33^

Even though depression, apathy, and loneliness were strong correlated, depression can be considered an affective disorder, apathy relates to disorders of motivation, and loneliness has a social dimension. Loneliness is a complex socioemotional trait with a specific construct that can be reliably measured^9^ and distinguished from affective disorders and behavioral symptoms. Consistently, about 30% of our patients experienced loneliness without having affective or behavioral disorders (Table 3). However, 60% of patients with behavioral impairment (45 ALSbi and 2 ALS‐FTD) reported feeling of loneliness. This association remained statistically significant also when controlled for age, mood, and motor dysfunctions (Tables 2, 3, 4) and deserve particular attention given the burden on patients and caregivers well‐being.^7^

Albeit inconsistently, loneliness was found to be a significant predictor of neuropsychiatric symptoms, including psychosis and depression, in people with dementia.^33^ In ALS, longitudinal studies are warranted to verify if loneliness can be considered a risk factor for the development of behavioral impairment. Our results indicated that loneliness may be a risk factor for severe symptoms of apathy and behavioral changes (Table 4).

### Emotional functioning and loneliness in ALS

We found that lonely individuals, both patients and HC volunteers, had difficulties in identifying and describing feelings, as shown by higher levels of alexithymia (Table 1), a cognitive‐affective disturbance characterized by dysfunction in emotional awareness.^34^ As documented by regression analyses (Table 4), we could infer that personal emotional knowledge can be influenced by the richness and strength of social connections. However, depressive mood may worsen alexithymic traits, whose facets defined by difficulties in identifying and communicating feelings are also related to depressive symptoms.^31^ Indeed, when controlling for the effect of mood, the association between the UCLA‐3L and the TAS‐20 scores did not survive Bonferroni correction (Table 2).

More robust were the findings concerning loneliness and emotional dysregulation, that is, the ability in monitoring, evaluating, and modifying emotional reactions to accomplish one's goals. We showed that patients with feelings of loneliness had limited access to emotion regulation strategies (Tables 2 and 4). Also in the general adult population, loneliness is associated with the increased use of unhelpful emotion regulation strategies.^35^ It has been suggested that sharing negative or positive emotions with others can downregulate stress or maximize the benefits of positive events.^36^ Therefore, the satisfaction of social connections can encourages the regulation of emotions. To achieve this, interdisciplinary approaches are needed to address the psychosocial needs of ALS patients and their families.

### Cognition and loneliness in ALS

In older adults, models adjusted for demographics, premorbid intelligence, depression, medical burden, and social network, demonstrated that loneliness has been prospectively associated with worsening performance on memory, executive functioning and processing speed,^37^ and with increased risk of incident dementia.^38^ In our study, the relationship between loneliness and cognitive efficiency was analyzed with partial correlations to control for the effect of age, education, mood, and functional motor disabilities. We could not evaluate the effect of premorbid IQ on such relationship, but patients with history of neurocognitive developmental disorder were not enrolled. We found that, in ALS patients, the global cognitive efficiency, measured with a screening test (ECAS) assessing several domains independently of ALS‐related motor dysfunctions, was not related to loneliness (Table 2). We speculate that patients' cognitive performances were not influenced by the satisfaction of social environment. This is in line with a recent meta‐analysis showing that poor social engagement is a robust risk factor for dementia and not just loneliness per se.^39^ From a cognitive perspective, we were also interested in verifying if loneliness was related to the reduced abilities in recognizing emotions and inferring others' mental and emotional states that have been associated to ALS.^34^ The hypothesis that loneliness may alter the way in which patients interpret the social world because of a reduce vigilance for social threat^40^ could only partially be confirmed in our cohort. Indeed, psychometric measures evaluating emotional empathy suggested that ALS/L+ patients might have diminished responses to other people's need and feelings than ALS/L− patients (Table S1), although their cognitive performances on tasks assessing emotion recognition abilities were similar to those of the ALS/L− (Tables 2 and 3). Nevertheless, it cannot be excluded that social isolation or social withdrawal, which often afflicts individuals with degenerative diseases, may affect patients' empathic and emotional recognition skills. Further studies focused on the several processes of maladaptive social functioning (such as the detection and processing of social stimuli, mentalizing, and social learning) are warranted to examine the social risk factors of cognitive decline in ALS.

### Cortical signatures of loneliness in ALS

Our exploratory analysis showed an involvement of frontoparietal regions in ALS/L+ compared to ALS/L− patients. Studies examining cortical signatures of loneliness in healthy individuals revealed altered structures and/or functions in the medial and dorsolateral portions of the prefrontal cortex, in anterior insula, amygdala, hippocampus, and posterior superior temporal cortex.^5^ These regions are thought to form the core of human “social brain” sub‐serving social abilities including perceptual, attentional, and affective processing of social information.^5^, ^41^, ^42^ Albeit ALS/L+ patients had thinner bilateral frontoparietal and left occipital cortices than ALS/L− (Fig. 2), we could not exclude that their thinning was due to other factors than loneliness. Indeed, brain regions associated with processing of social stimuli are often part of neural networks involved in higher order cognitive functions. However, regression analyses found an association of loneliness, together with age and behavioral change, with thinning of the right precuneus (Table S5). Future longitudinal study, with in‐depth assessment of social functioning, could advance current knowledge about the neurobiological basis of loneliness in ALS.

### Conclusion

This study has some limitations. The cross‐sectional design, and the lack of patients in the later stage of the disease, which hinder the analysis on how behavioral symptoms could further evolve in patients with feeling of loneliness. We could not verify if the lack of social networks or contacts might contribute to the behavioral profiles of ALS patients with feelings of loneliness. Despite loneliness is distinct from objective social isolation, loneliness and isolation are social determinants of older people's physical and mental health, QoL, and longevity. During COVID‐19 pandemic, they represented the most important adverse consequences of lockdown, affecting also ALS patients.^7^

In conclusion, our results indicated that feelings of loneliness may occur in a significant percentage of ALS patients and healthy individuals (up to 47%), and the higher the loneliness, the more severe the symptoms of depression and anxiety and the lower the QoL. Loneliness is not specific of ALS, but, in patients with ALS, loneliness might have a negative impact on affective and behavioral functioning irrespective of cognitive function and motor disability, with potential impact on patients' care and clinical trial design. Translating our results into novel research paradigms for ALS, we speculate that sociality, acting as a proxy of the “social‐relational” reserve, could promote personal resilience against behavioral impairment. The development of therapeutic interventions to manage the backlash of loneliness are warranted. Until then, monitoring social functioning could provide appropriate and timely care and support to patients with ALS and their families.

## Author contributions

Concept and design: MC, EDB, and GL. Acquisition, analysis, or interpretation of data: All authors. Drafting of the manuscript: MC, AT, EB, and AN. Critical revision of the manuscript for important intellectual content: All authors. Administrative, technical, or material support: MC, VF, AT, AN, MGB, GL. MC, and GL had full access to all of the data in the study and takes responsibility for the integrity of the data and the accuracy of the data analysis.

## Conflict of interest

The authors declare that the research was conducted in the absence of any commercial or financial relationships that could be construed as a potential conflict of interest.

## Supporting information


**Table S1.** Dimensional Apathy Scale (DAS) and loneliness.
**Table S2.** Difficulties in emotional regulation and loneliness. Differences in DERS subscores among ALS subgroups dived according to the presence of feelings of loneliness (ANOVA with age, education, and ALSFRS‐R as covariates) and partial correlations (correction for age, education, ALFRS‐R, and HADS‐MND total scores) between UCLA‐3L loneliness scale scores and FBI‐ALS subscores in the whole sample.
**Table S3.** Differences in clinical and neuropsychological measures among ALS subgroups performing MRI scans dived according to the presence of feelings of loneliness.
**Table S4.** Differences in brain cortical thickness values among ALS subgroups dived according to the presence of feelings of loneliness (ANCOVA with age as covariate) and partial correlations (correction for age, ALFRS‐R, and HADS‐MND total scores) between UCLA‐3L scores and cortical thickness values in the whole sample.
**Table S5.** Hierarchical regression analyses testing if loneliness, age, disease stage, and behavioral and cognitive profile can predict cortical thickness in ALS patients.
**Data S1.** Definition of brain regions of interest.

## Data Availability

The data that support the findings of this study are available at https://doi.org/10.5281/zenodo.6560317.
