# Supplementary material for: Loneliness as neurobehavioral issue in amyotrophic lateral sclerosis
Source: Ann Clin Transl Neurol. 2024 Feb 22;11(5):1122–34. doi: 10.1002/acn3.52028 (PMC11093239; doi:10.1002/acn3.52028)
Supplement: Supplementary file 1 — Table S1. Dimensional Apathy Scale (DAS) and loneliness. Table S2. Difficulties in emotional regulation and loneliness. Differences in DERS subscores among ALS subgroups dived according to the presence of feelings of loneliness (ANOVA with age, education, and ALSFRS‐R as covariates) and partial correlations (correction for age, education, ALFRS‐R, and HADS‐MND total scores) between UCLA‐3L loneliness scale scores and FBI‐ALS subscores in the whole sample. Table S3. Differences in clinical and neuropsychological measures among ALS subgroups performing MRI scans dived according to the presence of feelings of loneliness. Table S4. Differences in brain cortical thickness values among ALS subgroups dived according to the presence of feelings of loneliness (ANCOVA with age as covariate) and partial correlations (correction for age, ALFRS‐R, and HADS‐MND total scores) between UCLA‐3L scores and cortical thickness values in the whole sample. Table S5. Hierarchical regression analyses testing if loneliness, age, disease stage, and behavioral and cognitive profile can predict cortical thickness in ALS patients. Data S1. Definition of brain regions of interest. [file ACN3-11-1122-s001.docx]

**Loneliness as neurobehavioral issue in amyotrophic lateral sclerosis**

**Supplemental data**

| **Table e-1 Dimensional Apathy Scale (DAS) & loneliness** | | | | | | | | | | | | | |  |
| --- | --- | --- | --- | --- | --- | --- | --- | --- | --- | --- | --- | --- | --- | --- |
| **A) Differences in clinical and neuropsychological measures among ALS subgroups dived according to the presence of feelings of loneliness (ANOVA with age, education and ALSFRS-R as covariates) and partial correlations (correction for age, education, ALFRS-R and HADS-MND total scores) between UCLA-3L loneliness scale scores and executive apathy, emotional apathy and initiation apathy (corrected for the effect of age, mood, and motor-functional disabilities).** | | | | | | | | | | | | | |  |
|  | **Range** | | **No loneliness**  **ALS/L-**  **UCLA-3L = 3**  **(N=90)** | **Range** | | **Lonely ALS**  **ALS/L+**  **UCLA-3L ≥4 (N=56)** | | | **Group differences**  **F values (Sig.)** | **Partial Correlations coefficient (Sig.)*** | | |  |  |
| *Executive subscore* | 0-11 | | 2.63 (3.4) | 0-17 | | 5.25 (4.4) | | | **19.484 (<.001)** | **.349 (<.001); CI: .14; .53** | | |  |  |
| *Emotional subscore* | 0-15 | | 7.94 (3.3) | 3-23 | | 9.91 (4.2) | | | **10.062 (.002)** | **.369 (<.001); CI: .20; .52** | | |  |  |
| *Initiation subscore* | 0-24 | | 7.34 (3.4) | 0-22 | | 10.28 (5.2) | | | **10.049 (.002)** | **.257 (.002); CI: .06; .45** | | |  |  |
| **B) Contingency tables (percentages enclosed in parentheses) of the co-occurrence of loneliness (UCLA-3L score ≥ 1) and executive apathy, emotional apathy and initiation apathy. The p values of Fisher exact tests are reported.** | | | | | | | | | | | | | |  |
| ***Executive apathy*** | | ***Presence of apathy ^a^*** | | | ***Absence of apathy*** | | | Total | | | | p= 0.020 | | |
| UCLA-3L ≥ 4 | | 4 (3%) | | 52 (35%) | | | 56 (38%) | | | |  | | |  |
| UCLA-3L = 3 | | 0 (-) | | 90 (62%) | | | 90 (62%) | | | |  | | |  |
| Total | | 4 (3%) | | 142 (97 %) | | | 146 (100%) | | | |  | | |  |
| ***Emotional apathy*** | | ***Presence of apathy ^b^*** | | | ***Absence of apathy*** | | | Total | | | | p= 0.005 | | |
| UCLA-3L ≥ 4 | | 7 (5%) | | 49 (33%) | | | 56 (38%) | | | |  | | |  |
| UCLA-3L = 3 | | 1 (0.5%) | | 89 (61 %) | | | 90 (62%) | | | |  | | |  |
| Total | | 8 (5.5%) | | 138 (94.5%) | | | 146 (100%) | | | |  | | |  |
| ***Initiation apathy*** | | ***Presence of apathy ^c^*** | | | ***Absence of apathy*** | | | Total | | | | p= 0.005 | | |
| UCLA-3L ≥ 4 | | 11 (7%) | | 45 (31%) | | | 56 (38%) | | | |  | | |  |
| UCLA-3L = 3 | | 4 (3%) | | 86 (59%) | | | 90 (62%) | | | |  | | |  |
| Total | | 15 10(%) | | 131 (90%) | | | 146 (100%) | | | |  | | |  |
| Legend: a = DAS executive apathy cutoff ≥ 14; DAS emotional apathy cutoff ≥ 15; c = DAS initiation apathy cutoff ≥ 16. | | | | | | | | | | | | | |  |

| **Table e-2. Difficulties in emotional regulation & loneliness.**  Differences in DERS subscores among ALS subgroups dived according to the presence of feelings of loneliness (ANOVA with age, education and ALSFRS-R as covariates) and partial correlations (correction for age, education, ALFRS-R and HADS-MND total scores) between UCLA-3L loneliness scale scores and FBI-ALS subscores in the whole sample. | | | | | | | | | | | |
| --- | --- | --- | --- | --- | --- | --- | --- | --- | --- | --- | --- |
| ***DERS subscores*** | **Range** | **No loneliness**  **ALS/L-**  **UCLA-3L = 3**  **(N=109)** | **Range** | | **Lonely ALS**  **ALS/L+**  **UCLA≥4**  **(N=64)** | | **Group differences**  **F values (Sig.)** | | **Partial Correlations coefficient (Sig.)*** | |  |
| ***Non Acceptance*** | 5-27 | 10.14 (4.6) | 6-29 | 14.35 (5.3) | | **26.045 (<.001)** | | **.216 (.005); CI: .05; .39** | |  |  |
| ***Goals*** | 5-21 | 9.09 (3.7) | 5-25 | 11.4 (4.1) | | **15.123 (<.001)** | | .168 (.031); CI: .02; .30 | |  |  |
| ***Impulse*** | 6-21 | 8.34 (2.8) | 5-28 | 10.23 (4.4) | | **8.077 (.005)** | | .063 (.421); CI: - | |  |  |
| ***Awareness*** | 6-28 | 13.25 (4.4) | 7-27 | 15.12 (4.7) | | 4.729 (.031) | | .159 (.040); CI: .00; .31 | |  |  |
| ***Strategies*** | 8-23 | 11.79 (3.5) | 8-34 | 15.78 (6.1) | | **26.946 (<.001)** | | **.288 (<.001); CI: .11; .44** | |  |  |
| ***Clarity*** | 5-17 | 7.56 (2.5) | 5-19 | 10.14 (3.5) | | **21.495 (<.001)** | | .191 (.014); CI: .03; .35 | |  |  |
| Significant Correlations were bias corrected, and accelerated bootstrap 95% intervals were computed with 1000 bootstrap equally-sized samples obtained. Legend: ALSFRS-R = Amyotrophic Lateral Sclerosis Functional Rating Scale – Revised Version; CI = Bootstrapped Confidence Interval; DERS = Difficulties in emotional regulation scale; HADS-MND = Hospital Anxiety and Depression Scale for the use in Motor Neuron Disease. | | | | | | | | | | | |

| **Table e-3. Differences in clinical and neuropsychological measures among ALS subgroups performing MRI scans dived according to the presence of feelings of loneliness.** | | | | | | | | | |
| --- | --- | --- | --- | --- | --- | --- | --- | --- | --- |
|  | **No.** | **Range** | **ALS without loneliness**  **ALS/L-** | **No.** | **Range** | **Lonely ALS**  **ALS/L+** | **Group differences**  **T (Sig.)** | **Partial Correlations (Sig.)*** |  |
| **Loneliness** **(UCLA-3L)** | 45 | 3 | 3 (0) | 32 | 4-9 | 5.31 (1.5) | - | - |  |
| **Demographical and clinical data** | | | | | | | | | |
| Gender (male / female) | 45 | - | 19 / 26 | 32 | - | 13 / 19 | X^2^= 0.02 (1.0) |  |  |
| Years of Age | 45 | 24-81 | 56.26 (13.7) | 32 | 36-81 | 62.40 (11.2) | **T= -2.081 (.041)** | .101 (.385)**^a^** |  |
| Years of education | 45 | 5-25 | 13.53 (4.2) | 32 | 5-18 | 11.78 (3.6) | T= 1.908 (.060) | -.131 (.264)**^b^** |  |
| Months from symptom onset | 44 | 5-84 | 20.72 (14.8) | 32 | 2-72 | 26.45 (22.7) | T= -1.320 (.191) | .182 (.123) |  |
| Motor disability (ALSFRS-R) | 45 | 28-47 | 40.69 (4.6) | 31 | 26-47 | 39.36 (5.1) | T= 1.154 (.252) | -.076 (.525)**^c^** |  |
| King’s Clinical Stage  (1 / 2 / 3 / 4) | 45 | - | 22/12/10/1 | 32 | - | 13/11/6/1 | X^2^= 0.060 (.870) | - |  |
| Bulbar onset (Yes / No) | 45 | - | 7 / 38 | 32 | - | 7 / 25 | X^2^= 0.500 (.555) | - |  |
| Gene mutations (Yes/No/na)  C9orf72/SOD1/others | 45 | - | 9 / 35 / 1  (5 / 0 / 4) | 32 | - | 7 / 25 /0  (3 / 3 / 1) | X^2^= 0.020 (1.0) | - |  |
| ALScn / ALSimp | 45 | - | 26 / 19 | 32 | - | 13 / 19 | X^2^= 2.200 (.168) | - |  |
| **Neuropsychological data** | | | | | | | | | |
| Global Cognitive Efficiency (ECAS) | 45 | 60-127 | 107.48 (13.8) | 32 | 57-119 | 101.81 (16.2) | F= 0.773 (.382) | -.060 (.610) |  |
| Empathy (SET total score) | 41 | 7-18 | 14.31 (2.7) | 29 | 3-18 | 13.2 (2.7) | F= 0.757 (.387) | -.101 (.412) |  |
| Emotion recognition (Ekman) | 36 | 33-56 | 46.69 (5.7) | 23 | 34-52 | 44.61 (4.7) | F=1.041 (.312) | -.214 (.110) |  |
| Emotional regulation (DERS) | 41 | 37-96 | 60.07 (14.5) | 28 | 41-138 | 80.39 (22.2) | **F=16.68 (<.001)** | .309 (.011)  [.12; .50] |  |
| Behavioural change (FBI-ALS) | 45 | 0-15 | 1.73 (2.9) | 32 | 0-22 | 4.52 (5.0) | F= 8.265 (.005) | **.449 (<.001)**  **[.12; .68]** |  |
| Apathy (DAS) | 36 | 4-35 | 16.25 (8.0) | 26 | 5-48 | 23.23 (11.5) | **F= 11.839 (.001)** | **.309 (.002)**  **[.15; .58]** |  |
| Anxiety (HADS-MND) | 45 | 0-12 | 5.13 (3.5) | 32 | 0-16 | 5.96 (3.5) | F= 0.882 (.351) | .307 (.007)  [.15; .47] |  |
| State Anxiety (STAIY-1) | 43 | 22-55 | 41.88 (7.7) | 31 | 28-74 | 45.22 (9.6) | F= 2.883 (.094) | .034 (.778) |  |
| Trait Anxiety (STAIY-2) | 43 | 26-51 | 37.83 (5.5) | 31 | 30-60 | 46.03 (9.0) | **F= 18.34 (<.001)** | **.379(.001)**  **[.14; .57]** |  |
| Depression (HADS-MND) | 45 | 0-12 | 2.13 (2.3) | 32 | 0-12 | 3.93 (2.9) | F= 8.219 (.005) | **.331 (.003)**  **[.10; .56]** |  |
| Depression (BDI-II) | 44 | 0-18 | 6.36 (4.0) | 31 | 2-38 | 13.39 (9.4) | **F= 14.69 (<.001)** | **.438 (<.001)**  **[.24; .63]** |  |
| Alexithymia (TAS) | 42 | 28-67 | 41.09 (7.6) | 31 | 22-78 | 50.29 (13.6) | **F= 10.047 (.002)** | **.408 (<.001)**  **[.18; .60]** |  |
| Quality of life (WhoQol-Age) | 44 | 2-5 | 3.69 (0.5) | 30 | 1-5 | 3.22 (0.6) | **F= 10.192 (.002)** | -.339 (.004)  [-.52; -.15] |  |
| Caregiver burden (CBI) | 32 | 0-34 | 8.12 (9.2) | 26 | 0-33 | 10.50 (9.8) | F= 1.261 (.266) | **.417 (.001)**  **[.10; .65]** |  |
| Legend: ALScn = Cognitively Normal ALS; ALSFRS-r= revised Amyotrophic Lateral Sclerosis Functional Rating Scale; ALSimp = ALS with cognitive and/or behavioural impairment; BDI-II = Beck depression scale ECAS = Edinburgh Cognitive and Behavioural ALS Screen; SET=Story-based Empathy Task; HADS-MND = Hospital Anxiety and Depression Scale for use in Motor Neuron Disease; FBI-ALS = Frontal Behavioural Inventory – ALS version; DAS = Dimensional Apathy Scale; DERSF = Difficulties in Emotion Regulation Scale; n.a. = not available; UCLA-3L = University of California, Los Angeles loneliness scale – 3-Item version; WhoQol-Age = The World Health Organization Quality of Life in the Aging population; **Legend to apex**: ***** = correction for years of age, years of education, HADS-MND total scores and ALSFRS-R scores; **§** = between group differences were obtained with ANCOVA with years of age, education and ALFSRS-R as covariates; **a** = correction for years of education, HADS-MND total scores and ALSFRS-R scores; **b** = correction for years of age, and ALSFRS-R scores, and HADS-MND total scores; **c** = correction for years of age, years of education, and HADS-MND total scores; **c** = correction for years of age, years of education, and ALSFRS-R scores | | | | | | | | | |

**Appendix e-1 - Definition of brain regions of interest**

The brain regions of interest were selected from those previously described within the 'social brain network' or closely related to social isolation (perceived or not) and to the processing of social stimuli and social behaviors.1-5 The **table 5s** includes cortical thickness values (± standard deviation) of selected cortical regions.

1. Alcalá-López D, Smallwood J, Jefferies E, et al. Computing the Social Brain Connectome Across Systems and States. Cereb Cortex 2018;28(7):2207-32.

2. Düzel S, Drewelies J, Gerstorf D, et al. Structural Brain Correlates of Loneliness among Older Adults. Sci Rep. 2019;19;9:13569.

3. Lam JA, Murray ER, Yu KE, et al. Neurobiology of loneliness: a systematic review. Neuropsychopharmacology. 2021;46:1873-1887.

4. Li L, Bachevalier J, Hu X, et al. Topology of the Structural Social Brain Network in Typical Adults. Brain Connect. 2018;8:537-548.

5. Zovetti N, Rossetti MG, Perlini C, et al. Neuroimaging studies exploring the neural basis of social isolation. Epidemiol Psychiatr Sci. 2021;30:e29.

| **Table e-4: Differences in brain cortical thickness values among ALS subgroups dived according to the presence of feelings of loneliness (ANCOVA with age as covariate) and partial correlations (correction for age, ALFRS-R and HADS-MND total scores) between UCLA-3L scores and cortical thickness values in the whole sample.** | | | | |
| --- | --- | --- | --- | --- |
| **Region of Interest** | **No loneliness (N=45)**  **UCLA-3L = 3** | **Lonely ALS (N=32)**  **UCLA-3L ≥ 4** | **F (P value)**  **correction for age** | **Partial correlations (P value)** |
| **LEFT HEMISPHERE** |  |  |  |  |
| *caudal anterior cingulate* | 2.763 ± 0.35 | 2.678 ± 0.25 | 0.864 (.356) | -.014 (.904) |
| *caudal middle frontal* | 2.761 ± 0.20 | 2.717 ± 0.18 | 0.068 (.795) | .157 (.188) |
| *Cuneus* | 1.985 ± 0.16 | 1.883 ± 0.18 | **7.957 (.006)** | -.035 (.769) |
| *entorhinal* | 2.923 ± 0.45 | 3.031 ± 0.29 | 2.457 (.121) | .194 (.103) |
| *fusiform* | 2.861 ± 0.18 | 2.819 ± 0.15 | 0.607 (.438) | .089 (.458) |
| *inferior parietal* | 2.711 ± 0.17 | 2.615 ± 0.14 | **3.992 (.049)** | -.089 (.457) |
| *inferior temporal* | 2.851 ± 0.16 | 2.803 ± 0.14 | 1.058 (.307) | -.036 (.761) |
| *isthmus cingulate* | 2.330 ± 0.20 | 2.232 ± 0.17 | 3.057 (.085) | -.084 (.481) |
| *lateral orbito-frontal* | 2.804 ± 0.12 | 2.706 ± 0.17 | **6.939 (.010)** | -.092 (.444) |
| *Lingual* | 2.034 ± 0.11 | 1.947 ± 0.17 | **7.801 (.007)** | .007 (.952) |
| *medial orbito-frontal* | 2.561 ± 0.18 | 2.471 ± 0.19 | 2.767 (.100) | .008 (.946) |
| *middle temporal* | 2.997 ± 0.17 | 2.935 ± 0.15 | 0.847 (.360) | .055 (.649) |
| *parahippocampal* | 2.681 ± 0.29 | 2.648 ± 0.32 | 0.012 (.914) | .107 (.371) |
| *parsorbitalis* | 2.838 ± 0.18 | 2.741 ± 0.20 | 3.665 (.059) | -.065 (.590) |
| *posterior cingulate* | 2.643 ± 0.20 | 2.600 ± 0.20 | 0.583 (.481) | -.032 (.789) |
| *precuneus* | 2.613 ± 0.16 | 2.536 ± 0.16 | 2.141 (.148) | -.025 (.835) |
| *rostral anterior cingulate* | 2.882 ± 0.20 | 2.803 ± 0.20 | 1.849 (.178) | -.108 (.367) |
| *rostral middle frontal* | 2.666 ± 0.16 | 2.565 ± 0.13 | **6.316 (.014)** | -.123 (.303) |
| *Superior frontal* | 2.929 ± 0.18 | 2.847 ± 0.17 | 1.531 (.220) | .012 (.921) |
| *superior parietal* | 2.446 ± 0.15 | 2.370 ± 0.15 | 2.747 (.102) | -.009 (.942) |
| *superior temporal* | 2.919 ± 0.21 | 2.861 ± 0.17 | 0.198 (.658) | .084 (.481) |
| *transverse temporal* | 2.521 ± 0.26 | 2.436 ± 0.26 | 1.053 (.308) | .112 (.348) |
| *insula* | 3.026 ± 0.17 | 2.984 ± 0.19 | 0.622 (.433) | -.016 (.896) |
| **RIGHT HEMISPHERE** |  |  |  |  |
| *caudal anterior cingulate* | 2.679 ± 2.93 | 2.615 ± 0.26 | 0.298 (.587) | -.084 (.481) |
| *caudal middle frontal* | 2.771 ± 0.18 | 2.728 ± 0.15 | 0.386 (.536) | .105 (.380) |
| *cuneus* | 1.961 ± 0.151 | 1.911 ± 0.13 | 3.206 (.077) | .015 (.902) |
| *entorhinal* | 3.050 ± 0.35 | 2.973 ± 0.35 | 0.858 (.357) | .026 (.830) |
| *fusiform* | 2.864 ± 0.15 | 2.805 ± 0.14 | 1.836 (.180) | -.030 (.804) |
| *inferior parietal* | 2.695 ± 0.16 | 2.634 ± 0.11 | 1.963 (.165) | -.001 (.997) |
| *inferior temporal* | 2.875 ± 0.15 | 2.825 ± 0.14 | 1.996 (.162) | -.137 (.250) |
| *isthmus cingulate* | 2.448 ± 0.18 | 2.349 ± 0.14 | **5.103 (.027)** | -.193 (.105) |
| *lateral orbito-frontal* | 2.752 ± 0.12 | 2.677 ± 0.15 | **5.699 (.020)** | -.172 (.149) |
| *lingual* | 2.035 ± 0.13 | 1.972 ± 0.15 | 3.730 (.057) | .002 (.987) |
| *medial orbito-frontal* | 2.640 ± 0.14 | 2.553 ± 0.17 | **4.899 (.030)** | -.067 (.574) |
| *middle temporal* | 3.048 ± 0.15 | 2.977 ± 0.15 | 2.414 (.125) | -.014 (.909) |
| *parahippocampal* | 2.683 ± 0.23 | 2.619 ± 0.26 | 0.214 (.645) | .002 (.989) |
| *Pars orbitalis* | 2.902 ± 0.20 | 2.759 ± 0.18 | **7.448 (.008)** | -.173 (.147) |
| *posterior cingulate* | 2.671 ± 0.17 | 2.589 ± 0.16 | 2.213 (.141) | -.092 (.441) |
| *precuneus* | 2.611 ± 0.16 | 2.519 ± 0.13 | **4.259 (.043)** | -.137 (.250) |
| *rostral anterior cingulate* | 2.952 ± 0.47 | 2.927 ± 0.24 | 0.012 (.914) | .009 (.939) |
| *rostral middle frontal* | 2.671 ± 0.14 | 2.584 ± 0.12 | **5.438 (.022)** | -.143 (.230) |
| *superior frontal* | 2.960 ± 0.17 | 2.863 ± 0.15 | 3.179 (.079) | -.056 (.643) |
| *superior parietal* | 2.438 ± 0.13 | 2.337 ± 0.14 | **7.836 (.007)** | -.123 (.304) |
| *superior temporal* | 2.996 ± 0.21 | 2.910 ± 0.17 | 1.162 (.285) | -.006 (.959) |
| *transverse temporal* | 2.569 ± 0.26 | 2.451 ± 0.27 | 2.248 (.138) | .019 (.876) |
| *insula* | 3.058 ± 0.19 | 3.003 ± 0.19 | 1.136 (.290) | -.135 (.256) |

| **Table e-5: Hierarchical regression analyses testing if loneliness, age, disease stage, behavioral and cognitive profile can predict cortical thickness in ALS patients** | | | | | |
| --- | --- | --- | --- | --- | --- |
| **Dependent variables** | ***Constant & Independent Variables*** | ***B*** | ***Standard Error B*** | ***β*** | ***Sign. (2-tailed)*** |
| **Left Hemisphere** |  |  |  |  |  |
| **ROSTRAL-MIDDEL FROTNAL**  R = .301, adjusted R^2^ = .025  F_4.75_ = 1.377; p = 0.244 | - | - | - | - | - |
| **LATERAL-ORBITAL**  R = .289, adjusted R^2^ = -.018;  F_4.75_ = 1.274; p = 0.285 | - | - | - | - | - |
| **INFERIOR PARIETAL**  R = .424, adjusted R^2^ = .152  F_4.75_ = 3.067; p = 0.015 | Constant | 2.784 | .156 | - | <.001 |
|  | **Age** | -.004 | .001 | -.328 | **<.001** |
|  | King’s stage | .008 | .021 | .044 | .688 |
|  | FBI-ALS score | .002 | .005 | .041 | .744 |
|  | corrECAS total score | .001 | .001 | .136 | .231 |
|  | UCLA-3L score | -.018 | .014 | -.164 | .200 |
| **CUNEUS**  R = .204, adjusted R^2^ = -.028  F_4.75_ = 0.601; p = 0.699 | - | - | - | - | - |
| **LINGUAL GYRUS**  R = .148, adjusted R^2^ = -.048  F_4.75_ = 0.315; p = 0.902 | - | - | - | - | - |
| **Right Hemisphere** |  |  |  |  |  |
| **CINGULATE ISTHMUS**  R = .314, adjusted R^2^ = .035  F_4.75_ = 1.536; p = 0.190 | - | - | - | - | - |
| **PARS ORBITALIS**  R = .301, adjusted R^2^ = .026  F_4.75_ = 1.395; p = 0.237 | - | - | - | - | - |
| **LATERAL ORBITO-FRONTAL**  R = .290, adjusted R^2^ = .019  F_4.75_ = 1.286; p = 0.280 | - | - | - | - | - |
| **MEDIAL ORBITO-FRONTAL**  R = .276, adjusted R^2^ = .010  F_4.75_ = 1.151; p = 0.342 | - | - | - | - | - |
| **ROSTRAL-MIDDEL FROTNAL**  R = .337, adjusted R^2^ = .050  F_4.75_ = 1.792; p = 0.126 |  |  |  |  |  |
| **SUPERIOR PARIETAL**  R = .346, adjusted R^2^ = .057  F_4.75_ = 1.905; p = 0.104 | - | - | - | - | - |
| **PRECUNEUS**  R = .446, adjusted R^2^ = .142  F_4.75_ = 3.481; p = 0.007 | Constant | 2.788 | .149 | - | <.001 |
|  | **Age** | -.004 | .001 | -.315 | **.006** |
|  | King’s stage | -.008 | .020 | -.043 | .696 |
|  | **FBI-ALS score** | .012 | .005 | .317 | **.014** |
|  | corrECAS total score | .000 | .001 | .022 | .841 |
|  | **UCLA-3L score** | -.032 | .013 | -.304 | .**017** |
| Legend: corrECAS = Edinburgh Cognitive and Behavioural ALS Screen score corrected for age and education according to the Italian regression-based norms; FBI-ALS = Frontal Behavioural Inventory – ALS version; UCLA-3L = University of California, Los Angeles loneliness scale – 3-Item version | | | | | |
